# Danger signals – damaged-self recognition across the tree of life

**DOI:** 10.3389/fpls.2014.00578

**Published:** 2014-10-31

**Authors:** Martin Heil, Walter G. Land

**Affiliations:** ^1^Departamento de Ingeniería Genética, Centro de Investigación y de Estudios Avanzados del Instituto Politécnico Nacional-IrapuatoIrapuato, México; ^2^Molecular ImmunoRheumatology, INSERM UMR S1109, Laboratory of Excellence Transplantex, Faculty of Medicine, University of StrasbourgStrasbourg, France

**Keywords:** danger model, damage-associated molecular pattern, DAMP, immunity, wounding, injury, non-self

## Abstract

Multicellular organisms suffer injury and serve as hosts for microorganisms. Therefore, they require mechanisms to detect injury and to distinguish the self from the non-self and the harmless non-self (microbial mutualists and commensals) from the detrimental non-self (pathogens). Danger signals are “damage-associated molecular patterns” (DAMPs) that are released from the disrupted host tissue or exposed on stressed cells. Seemingly ubiquitous DAMPs are extracellular ATP or extracellular DNA, fragmented cell walls or extracellular matrices, and many other types of delocalized molecules and fragments of macromolecules that are released when pre-existing precursors come into contact with enzymes from which they are separated in the intact cell. Any kind of these DAMPs enable damaged-self recognition, inform the host on tissue disruption, initiate processes aimed at restoring homeostasis, such as sealing the wound, and prepare the adjacent tissues for the perception of invaders. In mammals, antigen-processing and -presenting cells such as dendritic cells mature to immunostimulatory cells after the perception of DAMPs, prime naïve T-cells and elicit a specific adaptive T-/B-cell immune response. We discuss molecules that serve as DAMPs in multiple organisms and their perception by pattern recognition receptors (PRRs). Ca^2+^-fluxes, membrane depolarization, the liberation of reactive oxygen species and mitogen-activated protein kinase (MAPK) signaling cascades are the ubiquitous molecular mechanisms that act downstream of the PRRs in organisms across the tree of life. Damaged-self recognition contains both homologous and analogous elements and is likely to have evolved in all eukaryotic kingdoms, because all organisms found the same solutions for the same problem: damage must be recognized without depending on enemy-derived molecules and responses to the non-self must be directed specifically against detrimental invaders.

## INTRODUCTION

Multicellular organisms across the tree of life share as common problems injury and infection, against which they must initiate immunity to maintain metabolic homeostasis and integrity. Research devoted to understanding immunity has traditionally focused on the detection of the non-self. For example, the “classical,” adaptive immune response in humans depends mainly on antibodies that serve as receptors of antigens stemming from pathogens or, in the case of transplantation, from the allograft (i.e., the genetically “foreign,” transplanted organ; Wood and Morris, 1995; Alan et al., 2001; Trinchieri and Sher, 2007). Similarly, attempts to understand the inducible responses in plants to herbivory or infection by pathogens generally focus on the detection of the non-self: specific prokaryotic molecules such as flagellin or chitin are perceived as microbe- (or pathogen-) associated molecular patterns (MAMPs/PAMPs), whereas molecules from the saliva, regurgitate or oviposition fluids of herbivores are perceived as herbivore-associated molecular patterns (HAMPs), to then mount adequate resistance responses (Jones and Dangl, 2006; Wu and Baldwin, 2010; Zipfel, 2014).

**FIGURE 1:**
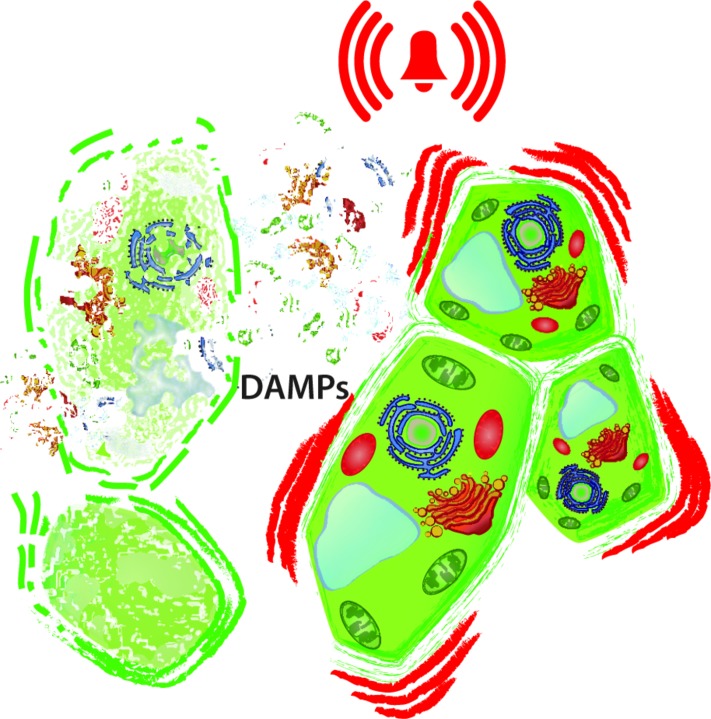
**Damaged-self recognition.** The disintegration of cells **(left)** releases intracellular molecules to the extracellular space and exposes macromolecules to hydrolytic enzymes from which they are separated in the intact cell. In principle, all these delocalised and newly produced molecules can serve as damage-associated molecular patterns (DAMPs) that prepare the neighboring, intact cell **(right)** for enemy recognition and wound sealing.

Specific responses to certain pathogens or herbivores evidently come with the advantage that they allow for highly targeted and, thus, energy-saving responses. However, we argue that this general model of immunity is incomplete as long as we ignore the mechanisms that organisms employ to monitor their integrity and to detect the “damaged self” (**Figure 1**; Matzinger, 2002; Heil, 2009; Land and Messmer, 2012). In general terms, an immune response cannot be based exclusively on the detection of the non-self, for the following reasons. First, pathogenic microorganisms and insect herbivores are way more diverse than their hosts. Thus, it appears difficult to imagine that a single host can evolve specific receptors to individually detect each of its potential enemies. In fact, there are more than one million species of arthropods described, the majority of which are considered herbivores, but we know only a handful of insect-derived elicitors of plant resistance responses (Wu and Baldwin, 2010). Second, all multicellular animals, plants, and fungi are exposed to the conspecific non-self, at least during sexual reproduction. Female organisms must tolerate invasion by pollen or sperm, which are genetically non-self, and females in most species carry the embryo for a certain time, which is 50% genetically non-self. Yet, neither mammals nor plants abort healthy embryos nor do fungi abort sporangia. Third, even intact, healthy multicellular organisms are colonized by microorganisms, i.e., representatives of the heterospecific non-self. Mammals (including humans) and other animals carry myriads of commensalistic or mutualistic microorganisms in their intestine (Turnbaugh et al., 2007; Kau et al., 2011), plants are regularly colonized by diverse endophytic bacteria, fungi, or viruses (Wilson, 1995; Arnold et al., 2000; Schulz and Boyle, 2005; Partida-Martinez and Heil, 2011), and even fungi can carry bacterial or viral endosymbionts (Partida-Martinez and Hertweck, 2005; Márquez et al., 2007; Partida-Martinez et al., 2007). How do hosts avoid uncontrolled immune responses that are triggered by these microbial associates? Finally, injury requires countermeasures that are completely independent of its causal reason. For example, any type of injury to the outer layers (such as skin, cuticle, or epidermis) of a multicellular organism promotes desiccation and pathogen invasion. Thus, organisms must be able to detect injury based on the perception of endogenous signals, rather than waiting for invaders to signal their presence. In summary, multicellular organisms must be able to detect wounding by perceiving endogenous “danger signals” (or “damage-associated molecular patterns,” DAMPs) and to elicit the corresponding general responses, including wound sealing and the induction of an altered state that allows for a fast and efficient detection of the non-self.

The model of a human immune system that is completely based on the perception of the non-self was challenged when Polly Matzinger and one of us (WGL) proposed the “danger hypothesis,” claiming that endogenous molecular signals of cell stress or injury play an important role in innate and adaptive immunity and in allograft rejection (Land et al., 1994; Matzinger, 1994). The model emerged from two independent sources. (1) The Land group employed data from a clinical trial in transplant patients that provided compelling evidence for immunity (here: alloimmunity-mediated allograft rejection) that is induced by tissue injury (here: allograft injury; Land et al., 1994). (2) Matzinger (1994) used a self-coherent chain of theoretical argumentation and concluded that the self/non-self discrimination theory of immune responses is incomplete. For plants, research efforts nowadays still focus on the detection of the non-self (i.e., HAMPs and PAMPs), although early studies used terms such as “wounding,” “wound response,” or “wound hormone” to denominate defensive responses to herbivory and the involved hormone, jasmonic acid (JA; Green and Ryan, 1972; Ryan, 1974; Graham et al., 1986; Stankovic and Davies, 1998; León et al., 2001).

Here, we first present a short overview on the danger model in mammalian immunology and then draw parallels to the current state of the art in plant science, which basically resembles the discussion that (human) immunology saw 20 years ago. We also review some of the major elements of the signaling cascade that are likely to play a role in the perception and transduction of wound-derived endogenous signals (DAMPs, or “danger signals”) across the tree of life. For example, eATP serves as danger signal and triggers immune responses in mammals (Chen and Nuñez, 2010; Zeiser et al., 2011; Gombault et al., 2013), fish (Kawate et al., 2009), insects (Moreno-Garcia et al., 2014), algae (Torres et al., 2008), plants (Demidchik et al., 2003; Chivasa et al., 2005), and fungi (Medina-Castellanos et al., data not shown). Similarly, fragments of the extracellular matrix are perceived as danger signals in organisms across the eukaryotes (Heil, 2012). Membrane depolarization events, Ca^2+^ influx into the cytosol, the formation of reactive oxygen species (ROS) and the transient phosphorylation of mitogen-activated protein kinases (MAPKs) have been reported during the first minutes in wounded or infected tissues of mammals, plants, fungi, and insects (see below). We finish with a short discussion of how likely these parallels are to represent homologies or rather the products of parallel evolution in unrelated organisms that are all under the same selective pressure: the need to reliably detect injury without depending on exogenous signals.

## THE DANGER MODEL IN MAMMALIAN INNATE AND ADAPTIVE IMMUNITY

**FIGURE 2:**
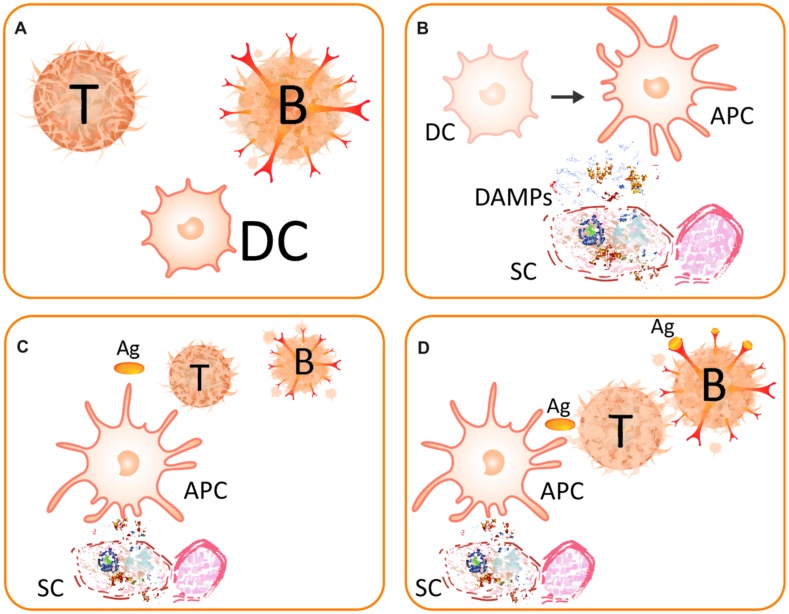
**The danger model. (A)** Main players of the (strongly simplified) danger model are the T-helper cell (T), the B-lymphocyte (B), and the dendritic cell (DC). **(B)** A somatic cell (SC) becomes destructed and releases DAMPs. The perception of these DAMPs causes the DC to mature to become an antigen-presenting cell (APC) and, thereby, gain immunostimulatory capacities. **(C)** An activated DC acts as APC and presents the antigen (Ag) to a naive T-cell. **(D)** The activated T-cell helps the B-lymphocyte, which thereby survives the recognition of the antigen. After Matzinger (2002).

In the mammalian immune system, two major layers can be distinguished: the innate and the adaptive immune response. In this context, “adaptive” refers to a phenotypic plasticity that optimizes an individual’s immune system for an acquired, highly specific, antibody-based response to current infection. Whereas the innate response is activated in response to the perception of DAMPs and PAMPs by receptors on pre-existing cells, a major characteristic element of the adaptive response is the proliferation of T- and B-lymphocytes (**Figure 2**) and their recruitment to the site of current infection. The proliferating B-lymphocytes are equipped with antibodies that very specifically target the antigens that are characteristic of the current invader (Wood and Morris, 1995; Trinchieri and Sher, 2007; Takeuchi and Akira, 2010), thus enabling a central feature of the mammalian immune system: the specific recognition of the invading non-self. Antigen presenting cells (APCs) such as dendritic cells (DCs) are required to stimulate T-helper cells and, consecutively, B-cells and, thus, translate innate immune events into adaptive immune processes. The danger/injury model claims that immunity is originally induced by tissue injury, rather than the presence of the non-self, and thereby adds an additional layer to the early recognition events (**Figure 2**). Because innate immune responses are simulated by damaged-self recognition, in the end both layers of the mammalian immune response are stimulated by DAMPs (Tamura et al., 2012).

After the discovery of innate immunity (Metchnikoff, 1908) and its re-discovery in the late 1990s of the last century (Lemaitre et al., 1996; Poltorak et al., 1998), the model of DAMP-triggered innate immunity was modified and extended (Land, 1999, 2003; Gallucci and Matzinger, 2001; Matzinger, 2002; Hirsiger et al., 2012). In general terms, it was proposed that endogenous molecules exposed on – or secreted by – stressed cells, or released from dying cells, are recognized by pattern recognition receptor (PRR)-bearing cells of the innate immune system. This recognition promotes inflammatory pathways, other parts of the innate immune system and eventually (in the presence of antigens) adaptive immune responses such as the activation on T-helper cells and B-lymphocytes (**Figure 2**) or the recruitment of neutrophils (Pittman and Kubes, 2013). Neutrophils are among the first leukocytes to be recruited from the bloodstream. Upon their activation by PAMPs and/or DAMPs, neutrophils follow directional cues, crawl along the vessel walls, exit the vasculature and move to the site of injury in the surrounding tissues (Pittman and Kubes, 2013). T-helper cells are required to support B-lymphocytes, which hypermutate to create new, potentially self-reactive cells and, thus, die if they recognize the antigen without help from active T-helper cells (Matzinger, 2002). These T-helper cells, in turn, require co-stimulation by activated DCs, which process the antigen and present it on their surface to T-cells. Hence, mature DCs act as APCs. In this context, sensing of DAMPs by PRR-bearing DCs promotes their maturation to APCs, which is associated with the acquisition of the capacity to elicit an adaptive immune response. Thus, APCs only co-stimulate T-helper cells when they are activated via PRRs such as Toll-like receptors (TLRs) that sense specific DAMPs (Land, 2003; Gallo and Gallucci, 2013).

Besides their direct contact with T-cells, activated DCs can also release specific cytokines such as interleukin 12 that help naïve CD4 cells to mature into active T-helper cells and, thereby, prime the immune system for the upcoming infection. In other words, the entire machinery that is required to recognize antigens and to mount an adaptive immune response is only activated when APCs, neutrophils, or macrophages sense DAMPs before. An intriguing example of the “raison d’être” of this complicated, multistep machinery is the manner by which epithelial cells of the intestine distinguish commensalistic from pathogenic bacteria. These cells respond to flagellin as a PAMP in a much stronger way when they are exposed to increased levels of eATP (Ivison et al., 2011). Here, the integration of DAMP perception into antigen recognition allows intestinal cells to distinguish damaging pathogens from commensals, which possess the same molecular signatures as pathogens but do not harm body cells. Clearly, the immune response needs active control to avoid collateral damage that might exceed the damage caused by pathogens (Zeiser et al., 2011). Molecular indicators of the destruction of body cells by pathogenic microorganisms are thus used in addition to their biochemical identifiers to distinguish between friends and foes in the human intestinal microflora.

## MAMMALIAN DAMPs AND THEIR PERCEPTION

The term DAMPs is differently used in the literature and can be replaced, for example, by terms such as “danger signals” or “alarmins.” For this review, we define DAMPs as cell-bound molecules or parts of macromolecules which are hidden from recognition by the immune system under normal physiological conditions. Under conditions of cellular stress or tissue injury, these molecules can either be actively secreted by stressed immune cells, exposed on stressed cells, or passively released into the extracellular environment from dying cells or from the damaged extracellular matrix (Hirsiger et al., 2012; Land, 2012; Gallo and Gallucci, 2013; Wenceslau et al., 2014). In the following, we only present some examples of mammalian DAMPs that represent the different classes, with a main emphasis on those examples for which equivalents have been detected in plants (**Table 1**). In mammals, DAMPs can even be of tissue-specific nature as, for example, crystals, and uromodulin molecules released by renal tubular damage represent kidney-specific DAMPs (Anders and Schaefer, 2014).

**Table 1:** Classification of mammalian damage-associated molecular patterns (DAMPs) based on their respective receptors and putative equivalents in plants.

Class of DAMPs	Mammals (Humans)	Cognate PRRs and perceiving cells	Equivalent DAMP in plants
Class I	HMGB1, HSPs, mtDNA, cytosolic RNA fibrinogen? biclycan?	TLR2, TLR3, TLR4, TLR7, TLR9, on macrophages, DCs and many somatic cells	Protein fragments such as systemin
Class II	ROS, eATP, cholesterol, uric acid	NLRP3 inflammasome I in macrophages, DCs and somatic cells	ROS, eATP
Class III	MIC A/B	Activating receptor NKGD2 and others (?), on innate lymphoid cells such as NK cells	
Class IV	Neoantigens such as NMHC-II, actin cytoskeleton, oxidized phospholipids	Pre-existing IgM antibodies → complement activation	Oligogalacturonides, oligosaccharides, pectin fragments
			Jasmonates
Class V	Perturbations of homeostasis, e.g., ER-stress		

*dsDNA, double-stranded DNA; eATP, extracellular ATP; ER, endoplasmatic reticulum; HMGB1, high mobility group box 1, an important chromatin protein; HSPs, heat shock proteins; MIC A/B, stress-induced soluble major histocompatibility complex class I-related chains A/B; mtDNA, mitochondria DNA; NK cells, natural killer cells, a type of “ready-to-go” cytotoxic lymphocyte critical to the innate immune system; NKGD2, natural killer group 2 member D; NLRP3, NOD-like receptor family, pyrin domain containing 3; NMHC-II, nonmuscle myosin class II protein; TLR, Toll-like receptor.*

**FIGURE 3:**
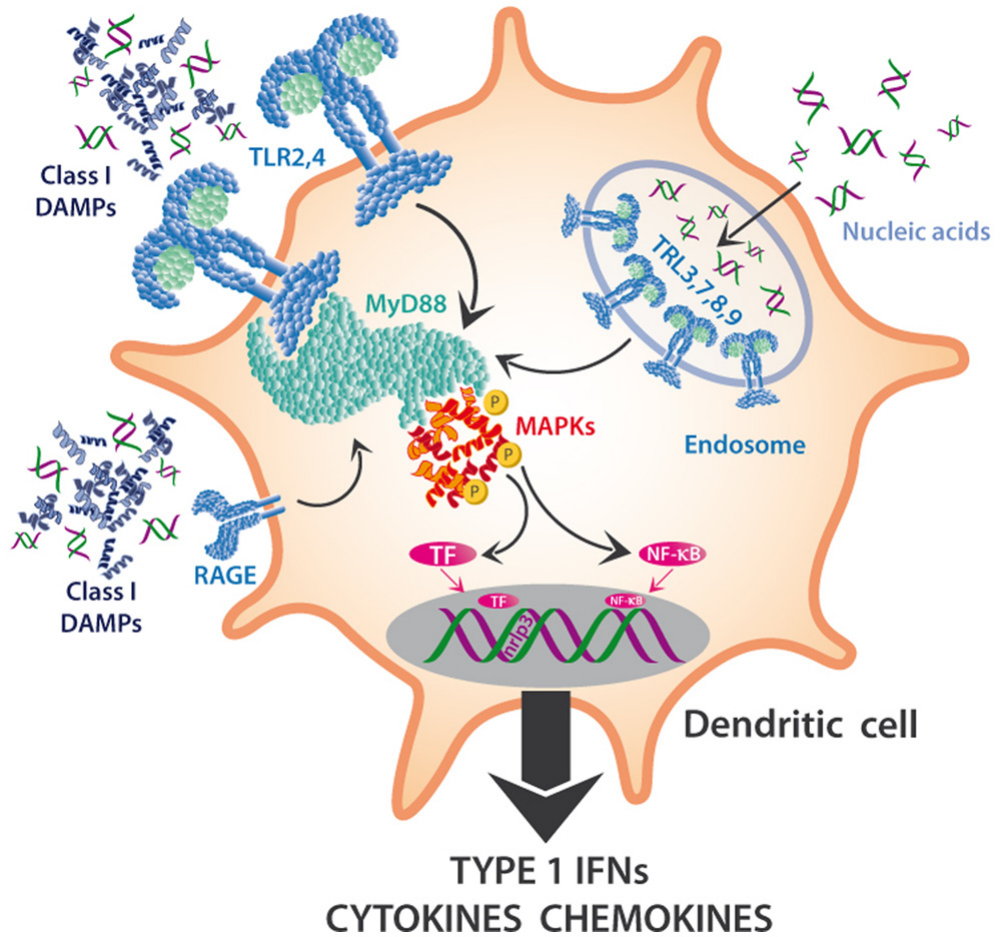
**MAPKs in the DAMP perception in innate immune cells.** DAMPs interact with multiple PRRs of innate immune cells and trigger the maturation of DC to mature APCs or the synthesis and release of Type I interferons (IFNs), cytokines, chemokines, and other pro-inflammatory compounds. Toll-like receptors (TLRs) such as TLR2 or TLR4 are located on the outer membrane, sense class I DAMPs (such as HMGB1) and initiate a pathway dependent on MyD88 and other mediators that triggers cascades that depend on mitogen-activated protein kinases, MAPKs (among others) and activate NF-κB and other transcription factors (TF). Nucleic acids can also be sensed via TLRs 3,7,8,9, which are located on the endosomal membrane, and activate the same downstream pathways.

We suggest to divide mammalian DAMPs into five classes (**Table 1**) because they are sensed by distinct members of five families of PRRs: TLRs (Kawai and Akira, 2010), receptor for advanced glycation endproducts (RAGE; Lee and Park, 2013), NOD-like receptors (NLRs; Zhong et al., 2013), RIG-I-like receptors (RLRs; Wu and Chen, 2014), and AIM2-like receptors (ALRs; Wu and Chen, 2014). Class I DAMPs comprise, for example, the important chromatin, high-mobility group protein B1 (HMGB1; Kang et al., 2014; Tsung et al., 2014), or heat shock proteins (HSPs; Seigneuric et al., 2011; Tamura et al., 2012), which are perceived via specific membrane-bound TLRs that act as PRRs and activate MAPK signaling cascades to induce inflammatory cytokines (**Figure 3**). MAPK signaling cascades are highly conserved elements in all eukaryotic cells that trigger the responses to multiple developmental or environmental stimuli. MAPK signaling cascades consist of three layers of kinases, in which MAPKs are activated via the simultaneous phosphorylation of a tyrosine residue and a threonine residue that are localized in an evolutionarily conserved “Thr-X-Tyr” motif in the activation loop of the MAPK. This phosphorylation is catalyzed by MAPK kinases (MAPPKs) that exhibit specificity both toward their MAPK and their respective upstream MAPKK kinase (MAPKKK). The latter type of enzymes forms a very diverse group of protein kinases that activate MAPKKs by serine/threonine phosphorylation, again in a conserved motif. Among others, MAPK cascades are involved in the perception of diverse DAMPs and PAMPs and, thus, represent central elements in the immune response to damage or infection (Kyriakis and Avruch, 2012).

Toll-like receptors are also involved in the perception of mitochondrial DNA (mtDNA; Zhang et al., 2010; Simmons et al., 2013) and of cytosolic double-stranded RNA (dsRNA; Amarante and Watanabe, 2010; Nellimarla and Mossman, 2014) and, again, activate downstream MAPK cascades. In DCs, TLRs that are located on the outer membrane sense class I DAMPs and initiate a pathway dependent on *Myeloid Differentiation Primary response gene* 88 (MyD88) and other mediators, which trigger MAPK cascades (Kyriakis and Avruch, 2012) and lead to the activation of NF-κB and other transcription factors (TF). Similarly, the capacity of eDNA to trigger the synthesis of complement factor B in macrophages in response to endogenous damage depends on HMGB1, MyD88 and NF-κB signaling (Kaczorowski et al., 2012), and the recently discovered DAMP S100A9 is perceived by TLR4 and mediates MyD88 signaling (Tsai et al., 2014).

Class II DAMPs such as ROS, monosodium ureate (MSU; Rock et al., 2013), eATP (Riteau et al., 2012; Gombault et al., 2013) or dsDNA (Patel et al., 2011) are sensed indirectly by the NLRP3 (*NOD-like receptor family protein 3*) inflammasome (see below) and, like class I DAMPs, are critical signals that are required for the maturation of DCs (Gallo and Gallucci, 2013).

Class III DAMPs comprise MIC-A, MIC-B (stress-induced soluble major histocompatibility complex class I-related chains A/B), and UL-binding proteins (ULBPs; Elsner et al., 2010; Li and Mariuzza, 2014; Nachmani et al., 2014) that are recognized by receptors such as NKG2D, an activating receptor that is expressed by innate lymphocytes such as NK cells and innate-like T-lymphocytes such as gamma delta T-cells.

Class IV DAMPs are defined here as neoantigens such as non-muscle myosin II (NMHC-II), actin cytoskeleton and oxidized phospholipids (Zhang et al., 2006a; Shi et al., 2009; Binder, 2012), all of which bind to pre-existing natural IgM antibodies to activate the complement cascade via the classical lectin receptors and alternative pathways.

Class V DAMPs or “Dyshomeostasis – Associated Molecular Patterns” refer to the recently described “homeostatic danger signals” (Gallo and Gallucci, 2013); they are defined here as an altered pattern of molecules reflecting perturbations in the steady-state of the intra- and/or extracellular microenvironment. These “homeostatic danger signals” include (but are not limited to) hypoxia, changes in acidity or osmolarity, and metabolic stress such as the accumulation of unfolded or misfolded proteins in the endoplasmatic reticulum (ER stress; Gallo and Gallucci, 2013; Garg and Agostinis, 2014).

## ACTIVATION OF THE NLRP3 INFLAMMASOME BY PAMPs, DAMPs, AND ROS

Priming is particularly pertinent to the activation of the NLRP3 inflammasome. The inflammasome is a multiprotein complex existing in innate immune cells such as DCs and macrophages; its exact composition depends on the activating factors and the cell type by which it is harbored. In its active form, the inflammasome is responsible for activation of inflammation and, eventually, programmed cell death. Multiple PAMPs and DAMPs activate the NLRP3 inflammasome, which contains NALPS (NACHT-, LRR-, and PYD-domains-containing protein 3), encoded by the *NLRP3* gene. Interestingly, class II DAMPs such as dsDNA can also interact with the class I DAMP, HMGB1 to form a complex that triggers a RGA-mediated activation of the inflammasome (Liu et al., 2014).

**FIGURE 4:**
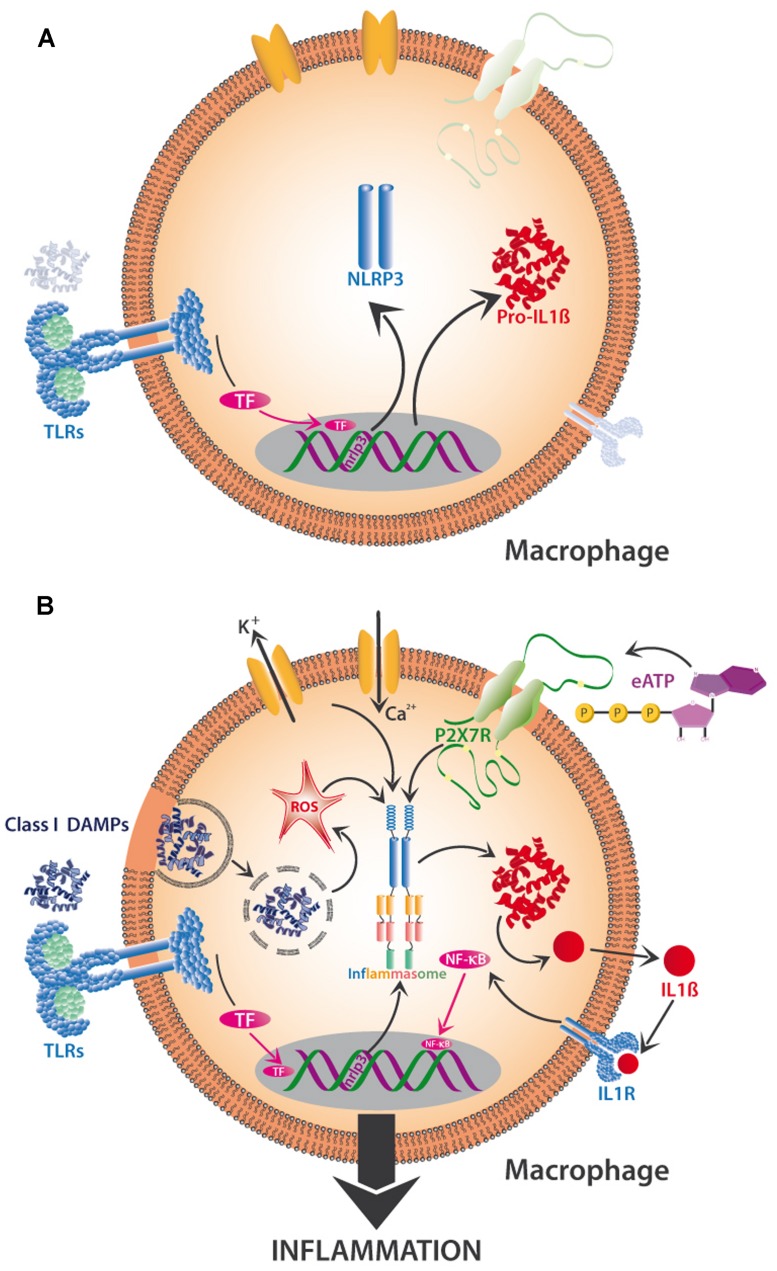
**Two-step activation by DAMPs of the NLRP3-inflammasome in mammalian macrophages.** The priming phase **(A)** is characterized by the perception of class I DAMPs such as HMGB1 by TLRs, which induces transcription-mediated up-regulation of the NLRP3 receptor (sensor!) and the synthesis of pro-interleukin 1β (pro-IL-1β). Finite activation of the inflammasome **(B)** occurs when DAMPs of various classes are directly or indirectly sensed by NLRP3. Among other mechanisms, the release of phagocytosed DAMPs from lysosomes and the resulting intracellular formation of reactive oxygen species (ROS), K^+^ eﬄux and Ca^2+^ influx, and the interaction of eATP with its receptor (P2X7R), all trigger assembly and activation of the inflammasome and subsequent synthesis of IL -1β, which interacts with interleukin receptor 1 (IL -1R) to activate TF such as NF-κB, resulting in the production of further proinflammatory substances to create full-scale tissue inflammation. See text for details.

Recent research indicates the existence of a priming step and a separate activation step that are required to trigger NLRP3 activity (**Figure 4**). When class I DAMPs such as HMGB1 or HSPs are sensed via TLRs on macrophages, they trigger the transcription-mediated up-regulation of the NLRP3 receptor, a response that can also be promoted by mitochondrial ROS. Besides the transcription-dependent recruitment of NLRP3, priming also includes the synthesis of the interleukin precursor, pro-IL-1ß (**Figure 4A**). Finite activation of the inflammasome is provided by class II DAMPs including cholesterol and uric acid crystals or by PAMPs, all of which can be taken up by phagocytosis and then released from lysosomes to trigger ROS-dependent NLRP3 assembly (**Figure 4B**). Alternatively, NLRP3 assembly can be triggered by K^+^ eﬄuxes and Ca^2+^ influxes or by the class II DAMP, eATP (Gombault et al., 2013), which affects NLRP3 via the activation of the P2X7 receptor (**Figure 4B**). In all cases, NLRP3 assembly triggers the production of IL-1β from pro-IL-1β (among other interleukins), its release from the cell, consecutive sensing via the interleukin receptor (IL-1R), activation of TF such as NF-kB and, finally, gene expression leading to inflammation or, ultimately, cell death (Tschopp and Schroder, 2010; Latz et al., 2013). In short, DAMP-triggered immunity contains a positive feedback loop (here: the upregulation of a DAMP receptor and of the substrate of interferon synthesis after a first exposure to DAMPs), which serves to prime the cell for a faster or stronger response once the stress is repeated. DAMP perception is involved in several parts of the activation process to avoid aberrant activation (**Figures 4A,B**).

## PLANT AND MAMMALIAN DAMPs

Plants possess no adaptive immune response and, thus, depend only on innate immunity (Jones and Dangl, 2006; Zipfel, 2014). Nevertheless, plants are resistant against most potential herbivores and pathogens and this resistance is due to a myriad of constitutive and inducible defense mechanisms that possess different degrees of specificity (Barrett and Heil, 2012). Induced resistance in plants against natural enemies is mainly controlled via two interacting signaling pathways (Pieterse et al., 2009). The octadecanoid signaling cascade, with the central hormone JA, is mainly directed against herbivores and necrotrophic pathogens (Wasternack, 2007; Campos et al., 2014), whereas biotrophic pathogens are controlled via responses that depend mainly on salicylic acid (SA; Métraux, 2001; Shah, 2003). Both pathways are usually subject to a negative crosstalk, due to which plants normally can mount resistance either to herbivores or to biotrophic pathogens, but not both at the same time (Thaler et al., 2012).

Early research into resistance to chewing herbivores such as beetles and caterpillars, termed the “plant wound response,” used leaf homogenate to elicit defensive responses: a treatment that applies indicators of the damaged self, rather than HAMPs as indicators of the non-self (Green and Ryan, 1972; Ryan, 1974; Turlings et al., 1993; Mattiacci et al., 1995). Disintegrated plants cells release DAMPs that can be sensed by as-yet intact cells and trigger defensive responses, just as we have described above for mammalian DAMPs (Heil, 2009, 2012). Plant DAMPs can be identical to human DAMPs, or represent functional equivalents (**Table 1**). For example, eATP induces multiple defensive responses in plants (Demidchik et al., 2003; Chivasa et al., 2005, 2009; Kim et al., 2006; Heil et al., 2012; Choi et al., 2014; Tanaka et al., 2014). Similarly, components of the human extracellular matrix serve as important DAMPs (Schaefer, 2010; Docherty and Godson, 2011; Zeiser et al., 2011), and cell wall-derived pectins, oligogalacturonides, and oligosaccharides represent some of the most classical inducers of plant defense responses (Doares et al., 1995; Creelman and Mullet, 1997; Stennis et al., 1998; Bergey et al., 1999; Orozco-Cardenas and Ryan, 1999; León et al., 2001). Pectin methylesterase releases methanol from the pectin in plant cell walls, and methanol acts as a potent volatile DAMP (Dorokhov et al., 2012; Komarova et al., 2014; Hann et al., 2014). It is likely the hydrolysis of cell-wall components that can also trigger JA-dependent defense responses to necrotrophic pathogens (i.e., pathogens that kill the cells of their host and feed on the content of the dead cells). Indeed, the release of oligomers from the polygalacturonate in *Arabidopsis* plant cell walls via a pectolytic enzyme from the soft-rot pathogen *Erwinia* sp. induced a gene that is involved in JA synthesis (Norman et al., 1999).

Fragments of human proteins such as collagen or fibronectin (Okamura et al., 2001; Thomas et al., 2007) find their equivalents in the high number of peptide signals in plants (Ryan and Pearce, 2003; Narváez-Vásquez et al., 2005; Chen et al., 2008; Yamaguchi et al., 2011; Albert, 2013; Bartels et al., 2013; Logemann et al., 2013; Ross et al., 2014), whereas the equivalents of the oxidized phospholipids that are considered as human class IV DAMPs (**Table 1**) are the oxidized lipids that constitute the octadecanoid signaling cascade: the central response in plants to damage caused by chewing herbivores (Schaller et al., 2005; Korneef and Pieterse, 2008; Pieterse et al., 2009). In fact, JA, a central hormone in systemic plant signaling, shows strong structural and biosynthetic homology to human prostaglandins (Ryan and Pearce, 1998; Wasternack, 2007).

Interestingly, the use of a programmable mechanical device (“Mec Worm”) that mimics the spatiotemporal feeding patterns of living herbivores caused lima bean (*Phaseolus lunatus*) to release a blend of volatile organic compounds (VOCs) that resembled what is seen after insect feeding on the same plant (Mithöfer et al., 2005). Similarly, the application of leaf homogenate to slightly damaged leaves of the same species caused an overall transcriptomic response that was very similar to the response to exogenous JA (Heil et al., 2012). Thus, it seems safe to assume that endogenous DAMPs are sufficient to elicit general plant resistance-related plant responses, at least when the DAMPs are applied/released at sufficient quantities and/or the correct composition. Among others, wounded plant cells release VOCs the earliest of which, called green-leaf volatiles (GLVs), are formed within seconds after injury (Scala et al., 2013). These VOCs can prime systemic parts of the locally damaged plants for future attack (Frost et al., 2007; Heil and Silva Bueno, 2007) and trigger resistance to herbivores and pathogens in neighboring plants (Heil and Karban, 2010), but many of them have also direct antimicrobial properties (Scala et al., 2013). As we discuss below, this double function as signals (at the afferent arc of an innate immune response) and direct antimicrobial agent (at the efferent arc) is a property of many DAMPs, for which reason we suggest that GLVs and other damage-induced plant VOCs represent a further class of DAMPs (Heil, 2014). However, we are not aware of volatile or gaseous equivalents in the known array of mammalian DAMPs.

## PLANT DAMPs AND THEIR PERCEPTION

**FIGURE 5:**
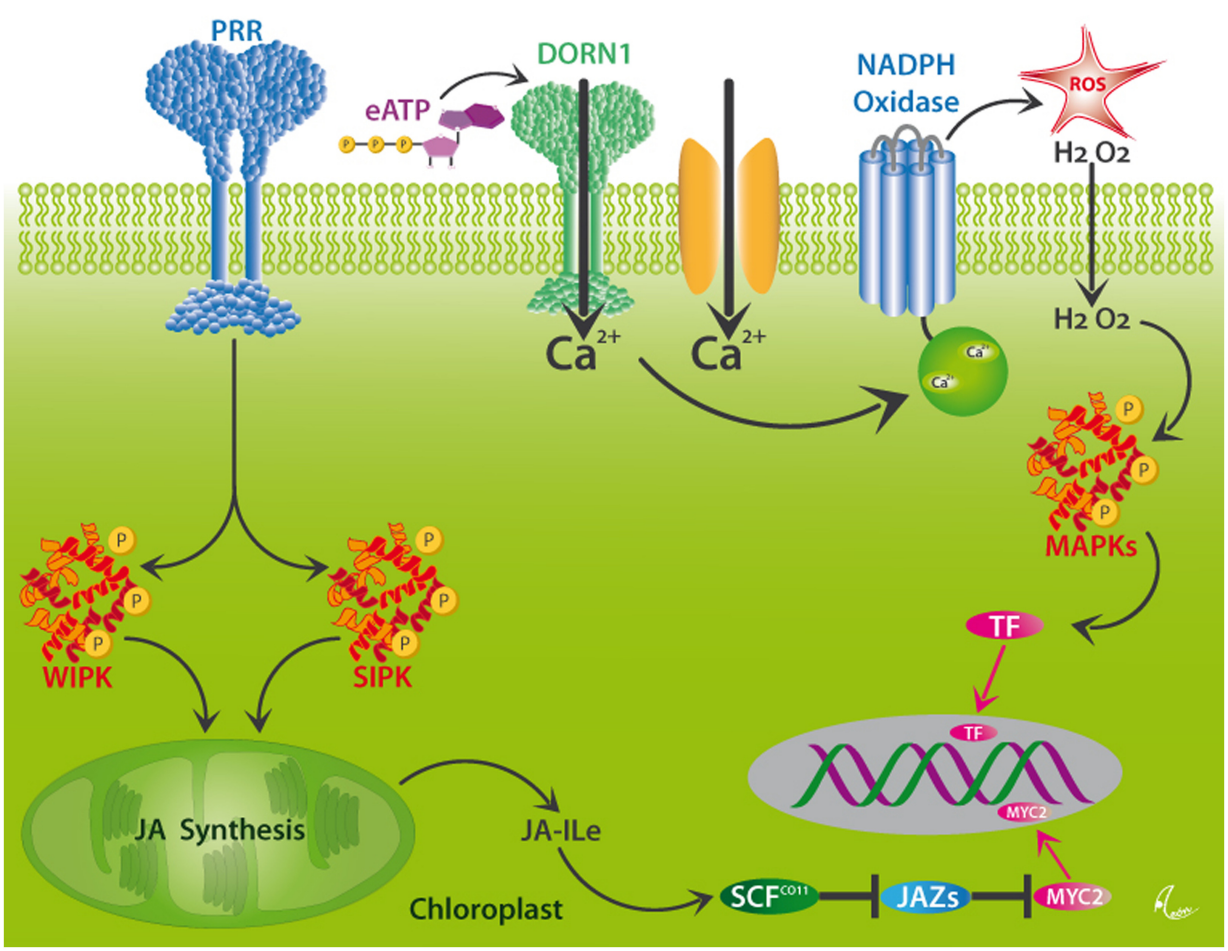
**Putative mechanisms for DAMP perception in plants.** Wounding activates the MAPKs, WIPK, and SIPK, likely via the perception of different DAMPs by as yet unknown pattern recognition receptors (PRRs). These kinases trigger the synthesis of jasmonic acid (JA) in the chloroplast. JA is conjugated to form JA-isoleucine (Ja-Ile), which interacts with its receptor, the F-box protein, COI1. JA-Ile specifically binds to COI1 protein and thereby promotes binding of COI1 to JASMONATE ZIM-DOMAIN (JAZ) proteins, which represent repressors of JA-induced responses in plants. This binding event facilitates the ubiquitination of JAZs by the SCF^COI1^ ubiquitin ligase, which leads to the subsequent degradation of JAZs and the release of TF, such as MYC2, and the consecutive expression of JA-responsive genes. Alternatively, Ca^2+^-influxes which can, among others be triggered by the perception of eATP by the DORN1 receptor, initiate the formation of ROS by NADPH oxidase, downstream MAPK signaling cascades and consecutive activation of the same genes via as-yet unknown TF. After Wu and Baldwin (2010).

In order to respond specifically to current attack, plants employ PRRs, the two most common classes of which are surface-localized receptor kinases (RKs) or receptor-like proteins (RLPs) that are commonly characterized by leucine-rich repeats (LRR) motifs. These PRRs perceive both PAMPs and DAMPs and, thus, play a central role in the resistance to pathogens (Zipfel, 2014). However, many plant DAMPs trigger both JA- and SA-mediated responses and, thus, are also involved in the resistance to herbivores (Duran-Flores and Heil, 2014; Ross et al., 2014). Hallmark steps in the perception of herbivory in plants are membrane depolarization events and the formation of electric signals (Maffei et al., 2004, 2007; Fromm and Lautner, 2007), Ca^2+^ influxes into the cytosol, the formation of ROS via a membrane-bound NADPH oxidase, and MAPK signaling cascades that ultimately activate TF and, thereby, the expression of resistance-related genes (León et al., 2001; Wu and Baldwin, 2010). Among the known plant MAPKs, the MAPK3/MAPK6 pathway is most commonly reported from the wound response in plants (Smékalová et al., 2014). Two further highly important MAPKs in this context are SA-induced protein kinase (SIPK) and wound-induced protein kinase (WIPK), which trigger the synthesis of JA from membrane-bound linolenic acid in the chloroplast and, thus, the octadecanoid signaling cascade (see **Figure 5**). In principle, all these steps could be activated via the perception of DAMPs by as-yet unknown receptors. Unfortunately, as to the very best of our knowledge, only few receptors for plant DAMPs have been characterized so far.

One of the most intensively studied plant DAMPs is systemin, a 18 amino acid polypeptide that upon wounding is processed from a 200-amino acid precursor called prosystemin, analogous to the functioning of peptide hormones in mammals (Ryan and Pearce, 1998). Interestingly, the systemin receptor in tomato is a transmembrane protein with LRRs on the extracellular surface, one transmembrane domain and a Thr/Ser kinase domain on the intracellular portion of the receptor (Scheer and Ryan, 2002). That is, it shares common motifs with the TLRs (Kawai and Akira, 2010, 2011) that perceive mammalian class I DAMPs. Recent research shows that systemin is only one example of a large class of small peptide molecules that trigger plant defense (Albert, 2013) and that usually are derived upon damage from precursors that play different roles in the intact tissue (Bartels et al., 2013). In *Arabidopsis thaliana*, small peptides (ATPeps) are perceived by LRR RKs (Bartels et al., 2013; Logemann et al., 2013), which indicates that the LRR-motif might be a common motif in the receptors of plant peptide DAMPs.

The receptor for eATP was discovered just earlier this year (Choi et al., 2014). The ATP-insensitive *Arabidopsis thaliana* mutant, *dorn1* (does not respond to nucleotides 1), was found to be defective in a lectin receptor kinase. DORN1 is a nucleotide-binding membrane protein with preferred affinity for ATP and is required for the eATP-induced calcium response. In *Arabidopsis*, Ca^2+^ influx triggers the development of ROS (Beneloujaephajri et al., 2013) and elevated levels of ROS activate MAPK3 and MAPK6 (Smékalová et al., 2014). Consequently, the mutant, *dorn1*, failed to trigger the phosphorylation of MAPK3 and MAPK6 (Choi et al., 2014). For *Arabidopsis*, eATP, Ca^2+^ influxes, ROS signaling, and the activation of MAPK3 were related to each other already in a study showing that levels of cytosolic free Ca^2+^ are determined by eATP perception at the plasma membrane and that eATP causes the production of ROS by plasma membrane-bound NADPH oxidase and the enhanced transcription of the MAPK3 gene (Demidchik et al., 2009).

Further receptors for plant DAMPs will have to be searched for in the future. However, circumstantial evidence from multiple plant species makes it tempting to speculate that plant DAMPs are generally perceived by and trigger the same signaling elements as they are known from the perception of PAMPs and HAMPs in plants, or from DAMP perception in mammals. For example, mechanical wounding or the application of conspecific leaf homogenates to common bean (*Phaseolus vulgaris*) caused the local development of ROS in the treated areas and the secretion of extrafloral nectar, which is a late, JA-dependent response to herbivory (Duran-Flores and Heil, 2014). The secretion of extrafloral nectar also increased after punching holes with a needle into the leaf blade of *Macaranga tanarius* (Heil et al., 2001), and sterile wounding enhanced the ROS levels in leaves of sweet potato, *Ipomoea batatas* (Rajendran et al., 2014). The same response was seen, for example, after soft mechanical stress in *Arabidopsis* (Benikhlef et al., 2013), or after using a razor blade to apply multiple sterile wounds to tomato (*Lycopersicon esculentum*) leaves (Orozco-Cardenas and Ryan, 1999). In fact, this form of mechanical wounding caused the generation of ROS in plant species in the Solanaceae, Poaceae, Cucurbitaceae, Fabaceae, and Malvaceae and required a functioning octadecanoid signaling cascade, at least in tomato (Orozco-Cardenas and Ryan, 1999).

Similarly, Ca^2+^ influxes from the apoplast into the cytosol are hallmark steps in the perception of DAMPs in mammals (**Figure 4**) and of HAMPs or PAMPs in plants (**Figure 5**), and Ca^2+^ influxes are also commonly observed after sterile, mechanical injury in plants (Leon et al., 1998; Beneloujaephajri et al., 2013; Benikhlef et al., 2013). In potato, a Ca-dependent protein kinase induces the development of ROS by NADPH oxidase, and NADPH oxidase appears to be a homolog of GP91^phox^, an oxidase from human phagocytes (Kobayashi et al., 2007). In maize, calcium-dependent protein kinase (ZmCPK11) triggers both local and systemic response to wounding (Szczegielniak et al., 2012). In summary, calcium signaling and enhanced levels of ROS have been discussed as a general component of the wound response in mammals and plants (Mittler et al., 2011; Ranf et al., 2011; Suzuki and Mittler, 2012) and both phenomena are likely to be triggered, at least in part, by the perception of DAMPs.

Mitogen-activated protein kinase cascades represent conserved signaling pathways in the response of eukaryotes to many types of environmental stress and play an important role in DAMP sensing in mammals (**Figure 3**; Kyriakis and Avruch, 2012). In the *Arabidopsis* genome, some 20 MAPKs and around 60 upstream MAPKKKs have been identified (Zhang and Klessig, 2001),which makes it tempting to speculate that MAPKs might act in the downstream signaling after the perception of DAMPs by as-yet unknown PRRs. In tobacco (*Nicotiana tabacum*), wounding alone activates SIPK and MIPK, although virus-derived PAMPs caused a stronger response (Zhang and Klessig, 1998). Silencing these two genes in wild tobacco, *N. attenuata*, confirmed that they are required for a complete response to wounding and downstream JA signaling (Wu et al., 2007). Application of HAMPs accelerated the wound response in this context, an observation that might have significantly slowed down the search for the DAMPs that must be involved in the responses of plant MAPK signaling to wounding. Indeed, the mutant of the *Arabidopsis* eATP receptor, *dorn1*, failed to trigger the phosphorylation of MAPK3 and MAPK6 (Choi et al., 2014), which represents a first case of a direct connection of DAMP perception to MAPK signaling in plants.

## DAMPs IN ALGAE AND FUNGI

Research into plant DAMPs has been slowed down because the research community focused on the handful of known insect- and pathogen-derived elicitors and their role in the perception of the non-self. Even less effort was devoted to deciphering wound recognition and downstream immunity-related responses in other organisms. Thus, we can only present scattered evidence from few systems here, which nevertheless makes us confident to conclude that wound recognition networks share common elements across the tree of life.

Both fungi and macroalgae respond to wounding with the formation of wound plugs that serve to seal the wound. This response can be elicited by mechanical, sterile wounding alone and, thus, clearly depends on the sensing of some kind of DAMP (Weissflog et al., 2008; Hernández-Oñate et al., 2012; Grosser et al., 2014). Unfortunately, we are not aware of many studies that investigated the early signaling events that lead to wound plug formation in algae. As mentioned above, eATP is a common DAMP and in fact, eATP also plays a role in the wound recognition in algae. For example, ATP is locally released from wounded cells of the alga, *Dasycladus vermicularis*, and experimental application of eATP to intact cells induced the production of H_2_O_2_ as an important downstream signaling response to wounding in this species (Torres et al., 2008). Similarly, wound-induced volatile compounds play a role in the wound response in the alga, *Dictyota dichotoma* (Wiesemeier et al., 2007), and waterborne signals can prime brown algae (*Laminaria digitata*) for faster responses to wounding or herbivore attack (Thomas et al., 2011), just as we have described above for GLVs, methanol and other VOCs that are released from plant wounds.

In the fungus, *Trichoderma atroviride,* mechanical wounding triggers Ca^2+^ influx and the production of ROS by a membrane-bound NADPH oxidase (Hernández-Oñate et al., 2012), and recent evidence now demonstrates that eATP can trigger the same responses and that downstream signaling is mediated via the MAPKs, Tmk1, and Tmk3, which represent the homologs of plant MAPK3 an MAPK6 (Medina-Castellanos et al., data not shown). Interestingly, some diffusible compounds of low molecular weight from fruiting body homogenate induced the development of fruiting bodies as a common wound response in the fungus, *Schizophyllum commune* (Rusmin and Leonard, 1978), which clearly hints to the involvement of DAMPs in the fungal wound response. In summary, the little evidence that we could find in this context makes it very tempting to speculate that basic steps via which macroalgae and multicellular fungi perceive wounding resemble those that we have described above for mammals and plants.

## POSITIVE FEEDBACK-LOOPS: DAMPs PRIME TISSUES FOR DAMP-RELEASE OR -PERCEPTION

As mentioned above, most researchers who studied the immune system in mammals or herbivore/pathogen-induced responses in plants focused on the detection of the non-self, whereas little effort was put into the active search for the endogenous danger signals. A further factor that might have hindered research into plant DAMPs is the frequency of seemingly contradictory observations concerning the early responses in plants to wounding. First, many studies report seemingly contradictory observations concerning the relative importance of DAMPs vs. PAMPs or HAMPs in the induction process, which might be in part simply due to the effect that several receptors, and TLRs in particular, interact with different molecules that comprise indicators of both, non-self and damaged-self (Escamilla-Tilch et al., 2013; Peri and Calabrese, 2013). The other way round, in mammals, a single DAMP such as HMBG1 can bind to different PRRs, e.g., TLR and RAGE (Ibrahim et al., 2013), and the same complexity and promiscuity of receptors might exist in plants as well. This high redundancy appears to be required to buffer the immune response against erroneous activation as well as invaders that mutate to achieve a stealthy mode of infection, but it poses significant problems on research as long as we search for linear chains that consist of one ligand, one receptor, and one downstream signaling element.

Furthermore, many potential plant DAMPs (such as systemin or cell wall fragments) appear to be localized both upstream and downstream of generally accepted signaling steps such as Ca^2+^ influx, the production of ROS, MAPK cascades and even the classical wound hormone, JA. In the following we discuss some examples of seemingly contradictory reports on signaling elements that are involved in the responses in plants to wounding.

First, a polygalacturonase releases oligogalacturonic acid fragments that trigger ROS production, and the expression of this polygalacturonase was found to respond systemically to local wounding or treatment with MeJA (Orozco-Cardenas and Ryan, 1999). In this scenario, the polygalacturonase appears to act downstream of wound hormone signaling. However, evidence from other studies indicated that oligogalacturonides elicit the formation of ROS (Stennis et al., 1998), thus characterizing their perception as an early event in plant wound signaling. Second, treatment with H_2_O_2_ can stimulate increases in cytosolic Ca^2+^ (Maffei et al., 2006), whereas enhanced cytosolic Ca^2+^ activated a potato NADPH oxidase via a Ca-depending protein kinase (Kobayashi et al., 2007) and also was found crucial to trigger the oxidative burst in *Arabidopsis* (Beneloujaephajri et al., 2013). Thus, Ca^2+^ influxes appeared downstream of ROS production in the first study, whereas in the other two studies, Ca^2+^ influxes were upstream of ROS production. Third, silencing both SIPK and WIPK in *N. attenuata* reduced the accumulation of JA after wounding, a reduction that was also reflected in the transcript levels of phytohormone biosynthetic genes and that would place MAPK signaling upstream of the synthesis of wound hormones (Wu et al., 2007). By contrast, a cascade consisting of MAPKK3 and MAPK6 was found to be activated by JA in *Arabidopsis* (Takahashi et al., 2007), an observation that would place MAPK signaling downstream of the octadecanoid cascade. Fourth, small peptides (Atpeps) represent an emerging class of DAMPs in plants and recent studies showed that the expression of the encoding PROPEP genes is induced when AtPeps are perceived by their corresponding receptors (PEPRs; Logemann et al., 2013; Ross et al., 2014).

In humans, the role of DAMPs in multiple diseases such as hypertension, various cancers, Alzheimer’s disease and diabetes is increasingly being appreciated and again, positive feedback loops represent a common phenomenon. For example, hypertension is associated with end-organ damage, leading to the release of DAMPs that trigger TLR-4 signaling. Since recent evidence suggests that TLR-4 signaling directly affects vascular contractility and, thus, blood pressure (Sollinger et al., 2014), the release of DAMPs appears both as a causal reason and as a consequence of hypertension (McCarthy et al., 2014). Similarly, sterile inflammation can be intensified by positive feedback-loops. For example, histones are released during sterile inflammation, act as DAMPs when they appear in the extracellular space, interact with TLRs to activate the NLRP3 inflammasome and, thereby, contribute to further cell death, which leads to the release of more DAMPs. Thus, it has been discussed that extracellular histones contribute to sepsis, small vessels vasculitis and acute liver, kidney, brain, and lung injury (Allam et al., 2014).

Positive feedback loops and network-like structures, rather than linear cascades, appear to be particularly characteristic of DAMP-mediated signaling and the associated resistance-related events. This positive feedback serves to prime the same cell (see **Figures 4A,B**) or the surrounding tissue for future injury or infection. In plants, for example, systemin triggers JA-dependent gene expression after wounding in the Solanaceae, and the expression of the gene that encodes prosystemin (the protein from which systemin is liberated after wounding) is induced by JA (Constabel et al., 1995; Ryan and Pearce, 1998; Pearce and Ryan, 2003). Thus, a first wounding event enhances the abundance of prosystemin and, thereby, prepares the plant to respond more strongly to future wounding. Systemin is involved both in the first and in the second part of this circle. This system shows astonishing similarity to the DAMP-induced transcription of the mammalian NLRP3 and Pro-IL-1β genes, which primes the macrophage for a faster and more intensive NLRP3-mediated perception of DAMPs and synthesis of IL-1β once it perceives further DAMPs or PAMPs (**Figures 4A,B**). Likewise, most of the genes that are involved in JA synthesis are JA-inducible and, hence, their expression is subject to positive feedback (Wasternack, 2007). Thus, the classical upstream-downstream model of signaling is likely not sufficient to understand the perception of DAMPs and the associated signaling events in plants.

## MULTIPLE ROLES OF DAMPs AS RESISTANCE INDUCERS, ANTIMICROBIAL AGENTS, AND MODULATORS OF REGENERATION

As discussed repeatedly (Dangl et al., 1996; Komarova et al., 2014), wounding is a strong predictor of infection and, therefore, the corresponding signaling must fulfill two non-exclusive functions: preventing infection and triggering the tissue for wound closure and other required responses. The ideal DAMP would fulfill all these functions and, surprisingly enough, many DAMPs have in fact been reported to function as antimicrobial agents and as resistance-inducing signals and/or exert a direct function in tissue regeneration. For example, mammalian type I interferons have direct antiviral activity and are also known for their immunomodulating properties (Gallucci and Matzinger, 2001), HMGB1 acts as class I DAMP, exerts cytokine-like activity (Lee et al., 2014) and stimulated the formation of regenerating fibers and vessel remodeling in the muscles in a mouse model (Campana et al., 2014). Kidney-specific DAMPs not only induce the NLRP3 inflammasome but also triger re-epithelializatian and contribute to the transition of epithelial to mesenchymal cells (Anders and Schaefer, 2014). In plants, DAMPs can trigger a set of basal responses such as cell wall strengthening, which are centrally involved in wound sealing (Delaunois et al., 2014), and ubiquitous DAMPs such as ROS have direct antimicrobial effects and also serve as signals (Dangl et al., 1996; Doke et al., 1996; Lamb and Dixon, 1997; Orozco-Cardenas and Ryan, 1999). Similarly, wound-induced methanol acts as anti-microbial agent and triggers defense responses in neighboring plants (Dorokhov et al., 2012; Komarova et al., 2014). This double function is likely to be a common trait of DAMPs, and in particular of plant VOCs that are released after cell damage. For example, nonanal has direct fungistatic effects (Zeringue et al., 1996) and the same compound induced resistance-gene expression in lima bean to *Pseudomonas syringae* (Yi et al., 2009). Similarly, methyl jasmonate inhibits the growth and aflatoxin production of *Aspergillus flavus* (Goodrich-Tanrikulu et al., 1995) and methyl salicylate has been shown to have antifungal activity against *Colletotrichum camelliae* (Zhang et al., 2006b); both VOCs represent the volatile forms of the resistance hormones, SA and JA, and, thus, their perception is likely to trigger resistance gene expression in most plants that are perceiving them. In general, many VOCs that are induced by herbivory or infection, and particularly GLVs, are well-known for their effects on defense expression, which can include both the direct induction of gene expression (Arimura et al., 2000) as well as its priming (Engelberth et al., 2004; Ton et al., 2007; Yi et al., 2009), and many GLVs and other plant VOCs also have direct antimicrobial effects (Dilantha-Fernando et al., 2005; Scala et al., 2013; Heil, 2014). In short, it is tempting to suggest that future research into potential plant DAMPs should particularly search for compounds that have direct antimicrobial (or, in the case of herbivores: repellent) effects and that also serve as signals that trigger gene expression in both the surrounding and in distant tissues, or organs. Cross-kingdom signaling might be common in this context and can be used by both, plant and plant enemy, for the manipulation of the other partner (Schultz, 2002).

## DAMPs PROVIDE THE BACKGROUND FOR PAMP/HAMP PERCEPTION

In spite of all the reports on wound-induced resistance responses in plants, there is a broad agreement across the botanical literature that HAMPs or PAMPs are required to elicit responses as they are seen after herbivore feeding or the infection by pathogens (see, e.g., Wu and Baldwin, 2010, and references cited therein). These seemingly contradictory points of view can be merged when we assume that DAMPs in plants play the same role as in mammals: as co-factors that prime as-yet healthy and intact cells for a full immune response, including the efficient perception of antigens (here: PAMPS and HAMPs). This hypothesis is also in line with the observation that the response to sterile damage in plants is usually correlated with the number of damaged cells: treatments such as the application of leaf homogenates, punching multiple holes with needles or squeezing leaves usually elicit detectable responses, whereas cutting off parts of the leaf blades or entire leaves with scissors or razor blades leaves few damaged cells on the plant and, thus, causes no response, or a response that remains below the detection limit (Heil, 2009).

In fact, most studies in plants found some response to wounding, but stronger responses to pathogen infection or herbivory (or to the application of the corresponding PAMPs or HAMPs into experimentally inflicted, sterile wounds). For example, sterile wounding activated MAPK signaling in tobacco spp., but much stronger responses were observed after the addition of virus particles or insect oral secretions (Zhang and Klessig, 1998; Wu et al., 2007). In an intriguing experiment, the “Mec Worm” device alone induced the formation of ROS but not electrical (V_m_) signals or Ca^2+^ influx, whereas the V_m_ response was as strong as seen after caterpillar-inflicted herbivory when caterpillar oral secretions (which contain fatty acid-amino acid conjugates as common HAMPs) were added to the mechanically damaged areas (Bricchi et al., 2010). Similarly, “Mec Worm” induced the release of VOC blends that were qualitatively identical to the herbivore-induced blends with respect to the major compounds (Mithöfer et al., 2005), whereas detailed analysis including minor components revealed that only adding oral secretions to the “Mec Worm”-inflicted damage caused spectra that were indistinguishable from the herbivore-induced ones (Bricchi et al., 2010). Finally, endogenous JA was enhanced after mechanical wounding of sweet potato, whereas feeding by herbivores induced both JA and SA (Rajendran et al., 2014). Thus, we hypothesize that DAMPs provide the necessary biochemical background for the successful perception of HAMPs and for the interpretation of MAMPs as PAMPs in plants and that high doses of chemically complex blends of DAMPs are enough to trigger resistance-responses on their own. This hypothesis is in line with the early observation that preincubation with systemin strongly enhanced the oxidative burst by which tomato cells respond to the exposure to oligogalacturonic acid fragments, which act as DAMPs in this system (Stennis et al., 1998). Tomato cells had been exposed to systemin 12 h before addition of the oligogalacturonides, which, in principle, gave the time for a transcription-based priming as we have described above for the effects of DAMPs in the mammalian NLRP3 inflammasome (**Figures 4A,B**). Studies aimed at further testing this hypothesis should add DAMPs, HAMPs, or PAMPs in different temporal orders to completely undamaged leaves (rather than adding them to slightly wounded tissues, which inevitably contain at least some DAMPs) and compare the responses to HAMPs or PAMPs in leaves that did, or did not, receive a pre-treatment with HAMPs.

## SIMILARITIES IN WOUND RECOGNITION ACROSS THE TREE OF LIFE: HOMOLOGIES OR OUTCOME OF ANALOGOUS DEVELOPMENTS?

In the above-listed examples we emphasized the similarities between DAMP perception in mammals and in other organisms, particularly plants. For example, the systemin-octadecanoid pathway has been characterized early as exhibiting “analogies to arachidonic acid/prostaglandin signaling in animals that leads to inflammatory and acute phase responses” (Ryan and Pearce, 1998) and DAMP-mediated TLR signaling is conserved in vertebrates and invertebrates (Ming et al., 2014). ER stress caused by unfolded or misfolded proteins in the endoplasmatic reticulum is a common trigger of inflammation and other immune responses in mammals (Gallo and Gallucci, 2013; Garg and Agostinis, 2014) whereas in plants, lacking quality control of protein folding in the ER equally can stimulate resistance resonses, likely via DAMP-mediated pathways (Tintor and Saijo, 2014).

Are these similarities evidence for homologies, that is: have central elements of wound recognition evolved before the diversion into plants, fungi and animals? Or do they present analogies, that is, independently evolved solutions of the same problems? Current evidence indicates that both answers are likely to be true. MAPKs represent a classical example of an evolutionarily conserved signaling cascade and multiple homologies have been found among MAPKs from mammals, plants and fungi (Zhang and Klessig, 2001; Hernández-Oñate et al., 2012; Kyriakis and Avruch, 2012; Smékalová et al., 2014; Medina-Castellanos et al., data not shown). Similarly, plant NADPH oxidase represents a homolog of gp91^phox^, an oxidase from mammalian phagocytes (Dangl et al., 1996; Kobayashi et al., 2007). In fact, even an inhibitor of the mammalian NADPH oxidase, diphenyleneiodonium, inhibits both the ROS burst and downstream signaling events in plants, and antisera to key mammalian proteins in this system cross-react with the respective plant proteins (Dwyer et al., 1995; Tenhaken et al., 1995). By contrast, the recently discovered receptor for eATP in plants (Choi et al., 2014) showed no sequence homology to mammalian P2X receptors, which are non-selective ATP-dependent calcium channels that are linked to the DAMP-dependent modulation of neurotransmitter release in neurons (Kucenas et al., 2003) and diverse physiological processes, including inflammation (Kawate et al., 2009; see also **Figure 4**). Thus, it appears that several elements of the wound recognition system are indeed that ancient that plants, fungi, and mammals share homologies in that respect, whereas other elements are functionally equivalent but have evolved independently as the functionally best solution of a problem that is shared by all multicellular organisms: reliable wound detection based on endogenous signals.

## CONCLUSION

The plant, fungal, and the mammalian immune systems are fundamentally different in two aspects. The mammalian system counts with specialized cells that can be equipped with specific sets of receptors and downstream signaling elements to fulfill their detailed function. Moreover, many of these cells are mobile and, thus, can be recruited to the site where their action is needed. By contrast, plants and fungal cells are encapsulated within rigid cell walls and, thus, each cell must be equipped to realize the entire immune response (León et al., 2001). Still, the early mechanisms by which wounding is perceived and downstream immunity-related signaling triggered comprise multiple similar elements. In part, these similarities represent homologies, whereas other parts represent the result of analogous evolution that responds with similar solutions to similar problems. The perception of endogenous danger signals, DAMPs that are released from the own, damaged tissue, represents a hallmark step that is present in all multicellular organisms. DAMPs can have antimicrobial and signaling activities and ensure that adequate responses such as wound sealing and defense against infection are induced almost instantaneously. DAMPs also prime for a more efficient perception of PAMPs/HAMPs and thereby allow to distinguish the harmless non-self (i.e., microbial mutualists and commensals) from the harmful non-self (i.e., pathogens) and to diminish the risk of an erroneous activation of immune responses, which would harm the organism itself. Unfortunately, the role of immunity classically was considered to be only the detection of the non-self. The research into the mammalian immune system has been dramatically slowed down by this narrow focus (Matzinger, 2002). Researchers who are interested in the immune response in plants, fungi or insects, thus, should try to widen the spectrum of signals for which they search and consider DAMPs as an important biochemical background for the perception of the non-self in multicellular organisms across the tree of life.

## Conflict of Interest Statement

The authors declare that the research was conducted in the absence of any commercial or financial relationships that could be construed as a potential conflict of interest.
